# Premature battery depletion of an insertable cardiac monitor: A 2-patient case series

**DOI:** 10.1016/j.hrcr.2026.07.030

**Published:** 2026-08-07

**Authors:** Chaitanya L. Malladi, Benjamin U. Hoffman, Kurt S. Hoffmayer

**Affiliations:** Section of Cardiac Electrophysiology, Division of Cardiology, University of California San Diego, La Jolla, California

**Keywords:** Insertable cardiac monitor, Implantable loop recorder, Battery depletion, Premature battery depletion, Remote monitoring, Device malfunction, Recommended replacement time

**Key Teaching Points**

• The Abbott Assert-IQ EL+ insertable cardiac monitor—rated for 6-year battery longevity—can undergo premature battery drain independent of transmission burden.
• The mechanism remains under investigation. Candidate explanations include battery cell defect, software-related power state dysregulation, and Bluetooth connectivity instability. These explanations are theoretical and unverified, and will require corroboration from engineering analysis of the explanted devices.
• Because accelerated depletion may occur without any clinically visible warning signal, direct battery percentage review at each remote transmission is warranted. An unexpected mid-course drop should prompt timely reporting to the manufacturer and, if the battery is low enough, evaluation for elective explant and re-implantation.

## Introduction

The Abbott Assert-IQ EL+ (DM5500; Abbott Medical, Sylmar, CA) is a Bluetooth-enabled insertable cardiac monitor (ICM) that received United States Food and Drug Administration clearance in 2023.^1^ Its rated 6-year battery longevity is the longest among currently available ICM devices.^1^ ICMs enable detection of clinically significant atrial and ventricular arrhythmias, including surveillance across post-ablation monitoring periods, long-term cryptogenic stroke evaluation, and atrial arrhythmia burden.^2^, ^3^, ^4^, ^5^

Battery failure in an ICM is clinically distinct from rapid battery depletion in a pacemaker or defibrillator. It imparts no hemodynamic consequence, but silently eliminates the diagnostic purpose of the implant and may necessitate unplanned reoperation.^2^ Premature battery depletion has not been previously reported for this device model in the literature or the Food and Drug Administration Manufacturer and User Facility Device Experience (MAUDE) Database. We present 2 patients at a single academic electrophysiology center whose Assert-IQ EL+ ICMs sustained premature battery depletion. Both devices achieved longevities substantially shorter than the rated 6-year lifespan, with a shared pattern of rapid mid-course battery decline not associated with device-based alerts or patient-initiated transmissions.

## Data sources and methods

All device data were obtained from remote monitoring transmission reports. Battery status in these reports is displayed as a graphical icon showing estimated remaining battery life as a percentage. Values were extracted from this icon at each available transmission timepoint and plotted chronologically to reconstruct the longitudinal depletion trajectory for each patient. Battery drain rates were calculated as the change in estimated battery percentage per day between consecutive transmission timepoints. Institutional review was consistent with applicable regulations governing case report publication at our institution.

## Case reports

### Patient 1

A 78-year-old man with atrioventricular nodal reentrant tachycardia status after slow pathway ablation and persistent atrial fibrillation status following cryoballoon pulmonary vein isolation underwent left parasternal implantation of an Abbott Assert-IQ EL+ ICM (DM5500; Abbott Medical) for atrial arrhythmia monitoring. Remote monitoring was established at the time of implant using the myMerlin™ (Sylmar, CA) mobile application and the patient’s Bluetooth-capable smartphone, not an Abbott mobile transmitter. Device settings were programmed to nominal for the implantation indication: atrial fibrillation (AF) monitoring.

Review of the battery trajectory from transmission reports (Figure 1) demonstrated a single episode of rapid battery decline. During the initial 17.5 months, battery charge declined at a mean rate of 0.06% per day, consistent with expected nominal drain for a 6-year device. Between 2 consecutive transmissions spanning approximately 35 days (days 533–568), battery charge fell from 62.6% to 9.7%: a drain rate of 1.51% per day and a 23-fold increase over the nominal phase. This interval was not associated with any device-based alert or patient-initiated transmission. Based on patient report and review of the electronic medical record, there was no known exposure to electromagnetic interference, radiation, or cardioversion during this period. Device-detected episodes matched transmitted episodes across our third-party remote monitoring platform, Vector Remote Care (Bend, OR) and myMerlin, with no discrepancy. Battery charge stabilized near 5%–10% thereafter, and recommended replacement time (RRT) was reached at approximately day 800, representing 26 months of total device function (36% of rated longevity). End-of-service was not formally reached, and the device continues to function at RRT. The patient was followed both remotely and in clinic as part of routine electrophysiology follow-up; at a clinic visit, the decision was made to electively explant and re-implant the device in the outpatient setting and to send the explanted device to Abbott Medical for engineering analysis.

**Figure 1:**
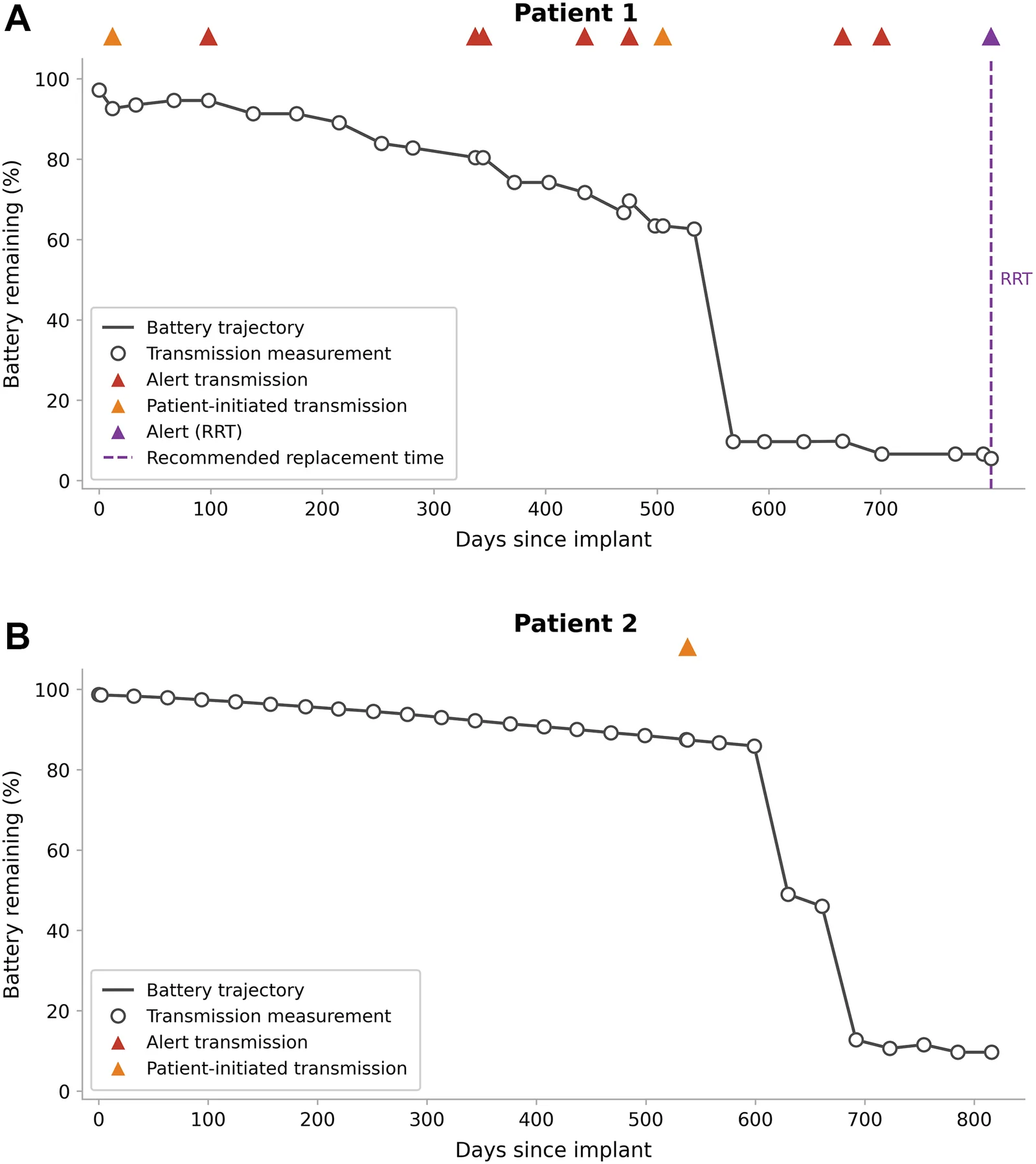
Battery trajectories for patients 1 (**A**) and 2 (**B**) following Assert-IQ EL+ ICM implantation. *Open circles* indicate all transmission measurements; *red triangles*, alert transmissions; *orange triangles*, patient-initiated transmissions; *purple dashed line*, recommended replacement time (RRT). In both patients, an initial period of nominal battery decline is followed by rapid depletion not associated with any transmission event.

### Patient 2

A 70-year-old man with a history of paroxysmal atrial fibrillation and atrial flutter status after catheter ablation underwent left parasternal implantation of an Abbott Assert-IQ EL+ ICM (DM5500; Abbott Medical) for post-ablation AF monitoring. Remote monitoring was established at the time of implant using the myMerlin™ mobile application and the patient’s Bluetooth-capable smartphone.

Unlike patient 1, patient 2’s ICM experienced 2 discrete episodes of rapid battery decline (Figure 1). During the initial 19.7 months, battery charge declined at a mean rate of 0.02% per day, well within the nominal range. On approximately day 599, a first episode of rapid decline occurred: battery charge decreased from 85.9% to 49.0% over 31 days (drain rate 1.19%/day; 55-fold increase over nominal). Battery charge then stabilized near 46%–49% for approximately 31 days. A second episode commenced at approximately day 661, with battery charge decreasing from 46.0% to 12.8% over 31 days (drain rate 1.07%/day; 49-fold increase over nominal). Neither period of rapid depletion correlated with a device-based alert or patient-initiated transmission. No device settings were changed before depletion. Based on patient report and review of the electronic medical record, there was no exposure to electromagnetic interference, radiation, or cardioversion. Device-detected episodes matched transmitted episodes on myMerlin and our third-party remote monitoring platform (Vector Remote Care), with no discrepancy. Battery charge has since stabilized near 9%–10%; RRT has not been triggered at the time of submission. As in patient 1, patient 2 was followed both remotely and in clinic as part of routine electrophysiology follow-up; at a clinic visit, the decision was made to electively explant and re-implant the device in the outpatient setting and to send the explanted device to Abbott Medical for engineering analysis.

## Discussion

Both patients experienced rapid mid-course battery depletion that was not associated with transmission burden. This pattern is distinct from depletion driven by excess electrogram storage or high transmission frequency, in which drain would be expected to scale with cumulative device usage.^1^ The culpable mechanism is under investigation by Abbott Medical. The candidate mechanisms below are theoretical explanations extrapolated from battery-depletion data in other cardiac implantable electronic devices. None has been verified or confirmed as causative in these cases, and diagnostic clarity awaits explant analysis by Abbott engineering.

### Battery cell defect

The sudden drop, which occurred after a prolonged period of stable function, may represent an internal battery defect. In published reports of premature implantable cardioverter-defibrillator (ICD) battery failure, lithium cluster formation produced an internal short circuit and sudden voltage collapse unrelated to device usage.^6^^,^^7^ A similar process could underlie the pattern observed here.

### Software-related power consumption

High transmission frequency alone has been shown not to compromise ICD battery longevity.^8^ However, an abnormal firmware-driven high-power or high-transmission state could in theory increase drain. For example, excessive communication between the ICM and the paired mobile device could increase power drain without any outward sign during standard transmissions. This has not been previously reported for this platform and remains theoretical, but would be potentially correctable by a software update.

### Bluetooth connectivity issue

A Bluetooth circuit component malfunction is a recognized failure mode in Abbott cardiac devices. A 2023 advisory identified a subset of Abbott ICDs and cardiac resynchronization therapy defibrillators in which this malfunction increased current consumption and substantially reduced device longevity; a subsequent firmware update mitigated the issue.^9^ That advisory does not include ICMs, but an analogous Bluetooth-related mechanism could contribute here. Because routine transmission logs do not capture background connection attempts, it is difficult to exclude and remains theoretical for this platform.

These explanations are not mutually exclusive. That 2 unrelated patients showed a similar pattern argues for a device-level rather than patient-specific contribution, although possible patient-specific factors were not specifically addressed in this report. Notably, neither patient was clearly exposed to electromagnetic interference or radiation, or underwent cardioversion, during the period of battery decline characterized here. Formal engineering analysis of the explanted devices will be essential to determine the root cause.

Both cases highlight an important phenomenon for clinicians to be aware of: battery status should be closely reviewed during routine remote interrogations, as rapid battery decline does not currently trigger an automated alert.

Two cases at a single center cannot establish the incidence of this problem, and the Assert-IQ EL+ otherwise represents a meaningful advance in ICM longevity. Notably, ICM manufacturers differ in how battery status is displayed on interrogation reports. Abbott displays an icon that visually represents the remaining battery percentage. Biotronik displays a coarser battery gauge (100%, 50%, and RRT), whereas Boston Scientific and Medtronic report a battery status indicator (eg, Good/OK, RRT). For this reason, granular characterization of mid-course battery drain described here cannot be fully replicated in non-Abbott platforms.

## Conclusion

Two cases of premature battery depletion of the Assert-IQ EL+ ICM, a device rated for 6-year longevity, are presented. In both cases, the battery dropped rapidly and well ahead of schedule, without adequate warning from standard remote monitoring alerts. The cause is under investigation by Abbott Medical. Until the mechanism is understood, clinicians should actively monitor battery percentage at each transmission and report similar cases through manufacturer complaint channels.

## Declaration of generative AI and AI-assisted technologies in the manuscript preparation process

During the preparation of this work, the authors used Claude (Anthropic, San Francisco, CA) to assist with manuscript drafting, editing, and figure preparation. After using this tool, the authors reviewed and edited the content as needed and take full responsibility for the content of the published article.

## Disclosures

All authors (C.L. Malladi, B.U. Hoffman, and K.S. Hoffmayer) declare that they have no conflicts of interest relevant to this manuscript. Abbott Medical had no role in the design, data analysis, or decision to submit this manuscript.
